# Supplementary material for: Improving the gnomonic approach with the gnomonicM R-package to estimate natural mortality throughout different life stages
Source: PeerJ. 2021 Apr 19;9:e11229. doi: 10.7717/peerj.11229 (PMC8061571; doi:10.7717/peerj.11229)
Supplement: Supplemental Information 1 — The value in parenthesis with asterisk refers to a difference in the estimation with respect to the original work. See (A) Ramírez-Rodríguez & Arreguín-Sánchez (2003), (B) Martínez-Aguilar, Arreguín-Sánchez & Morales-Bojórquez (2005), (C) Giménez-Hurtado, Arreguín-Sánchez & Lluch-Cota (2009), (D) Martínez-Aguilar et al. (2010), (E) Aranceta-Garza et al. (2016), (F) Romero-Gallardo et al. (2018). [file peerj-09-11229-s001.docx]

**Supplementary material**

| A) *Farfantopenaeus duorarum* | | |
| --- | --- | --- |
| Longevity = 480 days | | |
| MLF = 500,000 | | |
| G = 1.776 | | |
| 𝝰 = 1.615 | | |
| Stage of development | Duration (days) | $\bar{M}_{i}({year}^{-1})$ |
| Egg | 1.5 | 432.063 |
| Nauplius | 2.42 | 267.478 |
| Protozoa | 6.34 | 102.274 |
| Post-larvae | 16.57 | 39.106 |
| Juvenile | 43.34 | 14.952 |
| Pre-adult | 113.36 | 5.717 |
| Adult | 296.47 | 2.186 |

| B) *Sardinops caeruleus* | | |
| --- | --- | --- |
| Longevity = 2,555 days | | |
| MLF = [646,763 - 1,090,678] | | |
| G = 1.296 | | |
| 𝝰 = 1.159 (not reported numerically) | | |
| Stage of development | Duration (days) | $\bar{M}_{i}({day}^{-1})$ |
| Egg | **2.50** | 0.518 |
| Vit. Larvae | 2.90 | 0.447 |
| Early larvae | 6.26 | 0.207 |
| Late larvae | 13.52 | 0.096 |
| Early juvenile | 29.20 | 0.044 |
| Juvenile I | 63.07 | 0.021 |
| Prerecruit | 136.20 | 0.010 |
| Early adult | 294.15 | 0.004 |
| Adult | 635.26 | 0.002 |
| Late adult | 1,371.94 | 0.001 |

| C) *Epinephelus morio* | | |
| --- | --- | --- |
| Longevity = 7 300 days | | |
| MLF = [102,000 - 573,500] | | |
| G = 1.083 | | |
| 𝝰 = 1.271 | | |
| Stage of development | Duration (days) | $\bar{M}_{i}({year}^{-1})$ |
| Egg | **2.00** | 197.709 (**194.93*) |
| Larva, yolk vitellin | 2.54 | 155.546 (**153.10*) |
| Larva, preflexion | 5.77 | 68.490 (**67.35*) |
| Larva, flexion | 13.11 | 30.158 (**29.63*) |
| Larva, postflexion | 29.78 | 13.279 (**13.03*) |
| Transformed juvenile | 67.63 | 5.847 (**5.73*) |
| Benthic juvenile | 153.59 | 2.575 (**2.52*) |
| Subadult I | 348.80 | 1.134 (**1.12*) |
| Subadult II | 792.14 (**392*) | 0.499 |
| Adult I | 1,799.00 | 0.220 |
| Adult II | 4,085.65 | 0.097 |

| D) *Dodisicus gigas* | | |
| --- | --- | --- |
| Longevity = 438 days | | |
| MLF = [813,000 - 25,887,000] | | |
| G = 3.090 (**3.156*) | | |
| 𝝰 = 1.923 | | |
| Stage of development | Duration (days) | $\bar{M}_{i}({year}^{-1})$ |
| Egg | **6.00** | 188.000 (**299*) |
| Paralarva | 11.54 | 97.763 (**119*) |
| Juvenile | 33.73 | 33.446 (**41*) |
| Subadult | 98.58 | 11.442 (**14*) |
| Adult | 288.16 | 3.915 (**4.8*) |

| E) *Penaeus vannamei* | | |
| --- | --- | --- |
| Longevity = 365 days | | |
| MLF = 265,000 | | |
| G = 1.685 | | |
| 𝝰 = 1.962 (**4.027*) | | |
| Stage of development | Duration (days) | $\bar{M}_{i}({year}^{-1})$ |
| Egg | **0.54** | 1138.871 |
| Nauplius | 1.06 (**2.21*) | 580.334 (**278.28*) |
| Protozoa + Mysis | 3.14 (**9.25*) | 195.897 (**66.49*) |
| Post-larvae | 9.30 (**13.00*) | 66.127 (**47.31*) |
| Juvenile | 27.55 (**54.04*) | 22.322 (**11.38*) |
| Pre-adult | 81.62 (**72.96*) | 7.535 (**8.43*) |
| Adult | 241.79 (**213.00*) | 2.543 (**2.89*) |

| F) *Isostichopus badionotus*^+^ | | |
| --- | --- | --- |
| Longevity = 1,825 days | | |
| MLF = [13,500 - 5,062,490] | | |
| G = 2.286 | | |
| 𝝰 = 3.490 | | |
| Stage of development | Duration (days) | $\bar{M}_{i}({year}^{-1})$ |
| Early auricularia | **2.00** | 417.266 (**627.39*) |
| Medium auricularia | 6.98 | 119.558 (**418.26*) |
| Late auricularia | 31.34 | 26.627 (**209.13*) |
| Doliolaria-pentactula | 140.72 | 5.930 (**114.07*) |
| Juvenile | 631.86 | 1.321 (**0.69*) |
| Adult | 2,837.09 | 0.294 (**0.72*) |

^+^The estimated value of time duration for egg stage is not reported.
